# Stress levels of precursory strain localization subsequent to the crack damage threshold in brittle rock

**DOI:** 10.1371/journal.pone.0276214

**Published:** 2022-11-03

**Authors:** Özge Dinç Göğüş, Elif Avşar

**Affiliations:** 1 Istanbul Technical University, Geological Engineering, Istanbul, Turkey; 2 Konya Technical University, Geological Engineering, Konya, Turkey; Semnan University, ISLAMIC REPUBLIC OF IRAN

## Abstract

Micromechanical cracking processes in rocks directly control macro mechanical responses under compressive stresses. Understanding these micro-scale observations has paramount importance in predicting macro-field problems encountered in rock engineering. Here, our study aims to investigate the development of precursory damage zones resulting from microcracking pertinent to macro-scale rock failure. A series of laboratory tests and three-dimensional (3D) numerical experiments are conducted on andesite samples to reveal the characteristics of damage zones in the form of strain fields. Our results from discrete element methodology (DEM) predict that the crack damage threshold (*σ*_*cd*_) values are 61.50% and 67.44% of relevant peak stress under two different confining stresses (*σ*_*3*_ = 0.1 MPa and *σ*_*3*_ = 2 MPa), respectively. Our work evaluates the strain fields within the range of the *σ*_*cd*_ to the peak stress through discrete analysis for both confining stresses. We note that the representative strain field zones of failure are not observed as soon as the *σ*_*cd*_ is reached. Such localized zones develop approximately 88% of peak stress levels although the confinement value changes the precursory strain localization that appears at similar stress levels. Our results also show that the distinct strain field patterns developed prior to failure control the final size of the macro-damage zone as well as their orientation with respect to the loading direction (e.g 17° and 39°) at the post-failure stage. These findings help to account for many important aspects of precursory strain field analysis in rock mechanics where the damage was rarely quantified subtly.

## Introduction

Failure and deformation processes of rock samples in the laboratory have crucial similarities with natural macro-cracking (faulting). Both mesoscale and large-scale rock deformations are triggered by external stresses/loads and emanate from micro-interactions into the crustal domain [1–3]. Since the pioneering work of Griffith [4], understanding how and when microcracks nucleate, propagate and localize in rocks under stresses that cause rock deformation and damage is of great interest to researchers [3, 5–14]. Specifically, examining and comprehending these rocks’ micro-interactions at the pre-failure stage is paramount in predicting damage mechanisms.

Thanks to the acoustic emission (AE) technique in the laboratory, monitoring analyses can be conducted to observe the pre-failure microcrack accumulation [15–24] as well as to detect damage acceleration in brittle rocks consisting of pre-existing flaws [25–31]. Propagation and distribution of microcracks with increasing axial stress can be followed in rock samples by measuring the energy released. But in this technique, the characterization of damage by acoustic waves is sometimes difficult due to the low spatial resolution of the source location. Alternatively, X-ray tomography and digital image correlation methods have been integrated into laboratory experiments as higher resolution techniques for recording the incremental microcrack intensity in the rocks [32–40]. The amount and amplitude of microcracking in a material are directly detected during the loading. In addition, the spatial distributions of strain localization regions regarding microcracking can be resolved and described to display catastrophic rock failure.

Recently, numerical methodologies based on the discrete element methods (DEM) have been used to give an impetus for examining the transition from distributed microcracks to a macroscale crack under compressive loading conditions. The dynamics of microcracking in the form of localization zones and hence the progressive damage process into the rocks are captured in the DEM modeling platform. For instance, Wang et al. [41] studied the micromechanics of compaction localization zones in sandstones by discrete modeling simulations and suggested that shear localization transfers to distributed cataclastic flow with increasing stress. Shimizu et al. [42] developed a DEM code to reproduce the uncontrolled rock deformations (called Class II behavior) of brittle rocks at post-peak regions of the stress-strain curve. Class II behavior displays a form of localized deformation as a shear band emerging by microcracking in the model samples. Later, Schöpfer and Childs [43] investigated the elastoplastic behavior of porous rock and modes of localized deformation using the DEM technique. Model results suggested that average shear band inclinations, as the angle between the shear band normal and the loading direction, are a few degrees greater than predicted by localization theory in compression tests. Dinc and Scholtès [44] with their proposed 3D discrete modeling approach showed that strain localization results directly from shear microcracking developing with an orientation in the argillaceous rocks. Wu et al. [45] investigated the deformation bands in porous sandstones with a 2D hybrid DEM technique and suggested that shear-enhanced compaction bands and pure compaction bands are very similar in terms of microscopic characteristics. Later on, Zhang et al. [46] proposed a 3D DEM model to study the effects of anisotropy on the deformation and failure process of transversely isotropic rocks and pointed out that the localized bands are nearly parallel to the weak layers when the inclination angle of such layers is between 45° and 60° with respect to the loading direction. Dai et al. [47] examined the damage evolution related to microcracking in heterogeneous rocks by DEM model analysis and captured microcrack distribution that changes from diffusion to localization as stress increases. More recently, Zhang et al. [48] studied the permeability behaviors caused by cracks’ nucleation, propagation, and coalescence in low-permeable rocks. Their results derived from a DEM modeling approach show that permeability increases by several orders of magnitude afterward due to the appearance of a discrete strain localization band across the sample. Liu et al. [14] examined the characteristic changes in microcracks during progressive rock failure by building DEM models. Energy dispersion is not concentrated and the cracks do not localize when they emerge in the form of tensile cracks, contrary to the shear cracks’ occurrence. Overall, these numerical studies have supplied many useful insights into how the cracking process and hence the progressive damage develops in rocks. Nevertheless, to date, a precise description of strain (deformation) localizations in brittle rocks as precursory signals of a macro damage zone has not been clearly identified.

This study aims to numerically investigate localized deformation zones in a brittle rock regarding the microcracking process and to detect the critical stress levels of the rock damage under compression. In this context, a series of laboratory tests were initially conducted on brittle andesitic rock samples to obtain macro mechanical parameters. These parameters were then used in the calibration process of 3D numerical model samples. All numerical analyses were conducted through a 3D open-source DEM code called Yade Open DEM. During the simulations, microcracking is detected at different levels of quasi-static loading. Following this, progressive damage in the form of strain fields was calculated for both pre-and post-failure stages.

There are 3D numerical models in previous studies [e.g. 44, 46, 49, 50] identifying critical crack stress levels such as crack damage thresholds. However, the questions of “when or where is the damage forming before/after that point” and “what is the effect of the confining stress on this forming” have not been precisely and quantitatively answered yet. The novelty of this discrete modeling research is to reveal the explicit stress levels of the 3D strain localization regions subsequent to the crack damage threshold that can be considered precursory structures of a macro damage zone in brittle rocks.

## Materials and methods

In this study, both laboratory and numerical experimental methods are utilized. Due to representing a brittle rock behavior and homogenous texture, an andesitic rock- known as Ankara andesite in the literature- is selected as the material of this study. Laboratory experiments are firstly conducted on andesite core samples and then numerical experiments are set up based on the measurements obtained from these experiments. A 3D numerical DEM model is generated which is representing Ankara andesite in terms of its mechanical behavior. We present our methods in detail under the following sections; Laboratory Experiments, and Numerical Experiments associated with the section Model Parameter Calibration.

### Laboratory experiments

A series of uniaxial and triaxial compressive strength tests and splitting tensile tests were performed to obtain the macro mechanical parameters (uniaxial compressive strength, *UCS*; uniaxial tensile strength, *UTS*; Young’s modulus, *E*; and Poisson’s ratio, *v*) of andesite in the laboratory. All core sampling studies were performed by the procedures of ASTM D4543 [51] and prepared to be 54 mm in diameter with length-to-diameter ratios of 2.0 to 2.5. A diamond core barrel as being compatible with hard rocks was used to extract core samples from the andesite blocks.

**Fig 1:**
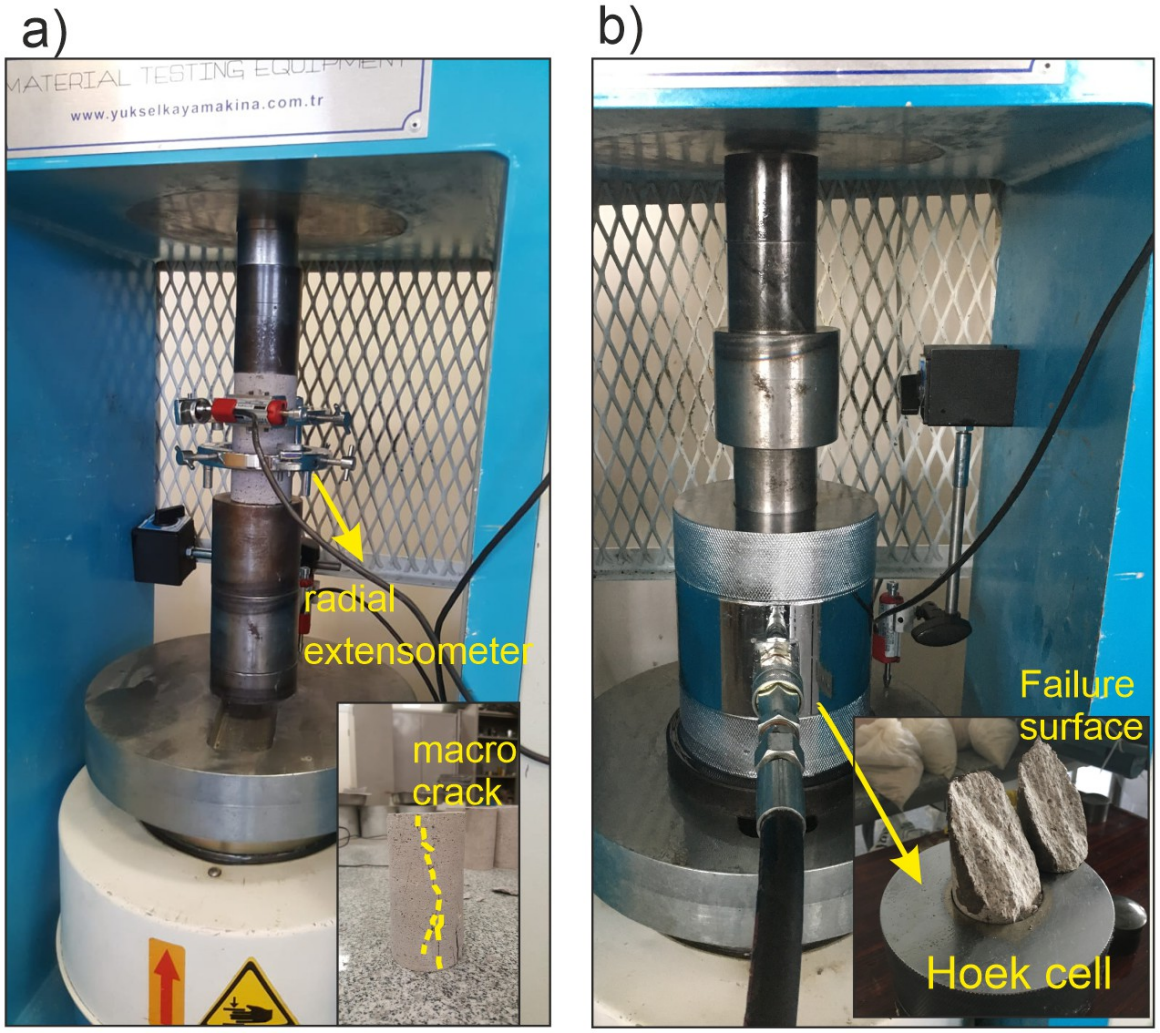
Implementation of laboratory tests under compressive loading conditions. (a) Uniaxial compressive strength test (b) Triaxial compressive strength test.

According to the method suggested by ASTM [51], ten uniaxial compressive strength tests were performed to obtain the *UCS*, *E*, and *v* of the samples. A loading frame with a maximum loading capacity of 1000 kN, a data logger, and a radial extensometer were used in these tests (Fig 1a). The axial load was arranged to increase continuously at a constant strain rate of 3x10^-3^ mm/s and applied to samples until the complete failure of the rock material. As a result of these measurements, the *UCS* values are within the range of 85.5 to 99.2 MPa. Lateral and vertical deformations that occurred during the *UCS* tests were recorded through linear variable differential transformers (LVDTs) to calculate the *E* (tangential modulus) and v of the andesite. The values of *E* and *v* are within the range of 10 to 12.5 GPa and 0.17 to 0.25, respectively.

Sixteen triaxial compressive strength tests were carried out on the core samples under the confining stresses of 2, 5, 10, and 14 MPa to determine the failure envelope of the andesite. The loading frame used in the *UCS* tests was now integrated with a pumping unit and a Hoek’s cell to arrange the confining stress (Fig 1b). Once the relevant confining stress was reached, the axial load was implemented with a constant strain rate of 5x10^-3^ mm/s.

For determining the *UTS* of andesite, disk shape samples were prepared for the splitting tensile test based on ASTM D3967-16 [51] testing procedures. The ratios of the thickness to diameter (*t/D*) of the samples are between 0.45 and 0.60. From the tests, the *UTS* values are calculated within the range of 7.5 to 11.2 MPa. As a result of the measurements, the ratio of *UCS*/*UTS* of andesite rock samples is approximately ≈10 characterizing the brittle rock response. All parameters obtained from the laboratory work are listed in Table 1.

**Table 1:** Macro mechanical properties of andesite obtained from laboratory measurements and DEM simulations.

Parameter	*UCS* (MPa)	*UTS* (MPa)	*E* (GPa)	*v* (-)
**Laboratory**	91.3 ± 5	9.2 ± 1	11 ± 2	0.18 ± 0.04
**DEM**	86	8.9	12.5	0.11

### Numerical experiments

We performed the numerical experiments using a three-dimensional open source software called Yade [52] based on the DEM. The modified version of the bonded particle model (BPM) of Potyondy and Cundall [53] was integrated into the DEM platform [54].

**Fig 2:**
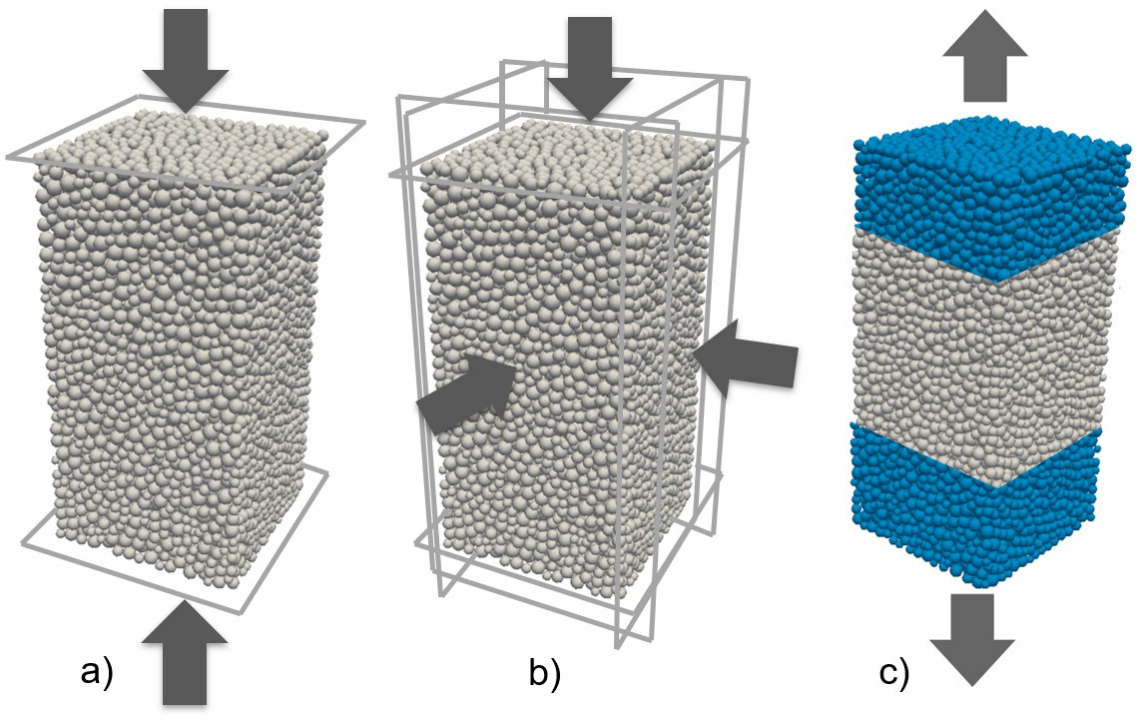
Numerical model samples. (a) Uniaxial compressive (b) Triaxial compressive (c) Direct tensile test configurations (The arrows show the loading directions during the simulations).

The rock material is represented by an assembly of rigid and spherical particles bonded together in polydisperse distribution (Fig 2). Particles -called also discrete elements (DEs)-interact with each other based on the elastic-brittle contact law. When the packing of the particles generates the numerical model sample, pairs of initially interacting DEs are identified within an interaction range, *γ*_int_, such that

$$D_{eq}\leq \gamma_{int}*(R_{x}+R_{y})$$ (1)

where *D*_*eq*_ is the initial equilibrium distance and, *R*_*x*_ and *R*_*y*_ are the radii of particles x and y. *γ*_*int*_ controls the initial number of interacting bonds, irrespective of the particle numbers in a packing. This parameter (*γ*_*int*_) arranges the average number of bonds per particle, *N* (coordination number), and can be defined before the simulation starts. Thus, it is possible to simulate the failure behavior of all types of rocks from soft to hard in the right strength ratio (*UCS* / *UTS*). For instance, when *γ*_*int*_ is close to 1, particles’ interlocking degree decreases, indicating a weak rock material. A critical point is that *γ*_*int*_ cannot be greater than the relative distribution of particle diameters inside the assembly. For example, in this study, *R*_*max*_ / *R*_*min*_ = 2, so that *γ*_*int*_ could not be greater than 1.5 (see [54] for details).

The interaction forces between the particles are subdivided into a normal component *F*_*n*_ and a shear component *F*_*s*_. In the normal direction, *F*_*n*_ is computed as:

$$F_{n}=k_{n}*u_{n}$$ (2)

$$k_{n}=2Y*\frac{R_{x}*R_{y}}{R_{x}+R_{y}}$$ (3)

where *k*_*n*_ is the normal stiffness as a function of equivalent elastic modulus, *Y* (in Pa) and u_n_ is the normal relative displacement.

Under compressive loading, *F*_*n*_ is not restricted and can increase indefinitely. In tension, F_n_ can increase up to the threshold value *F*_*n*,*max*_ = *t* * *A*_*int*_ computed from the interparticle tensile strength *t* (in Pa) and a surface *A*_*int*_ = *π* * [*min*(*R*_*X*_;*R*_*y*_)^2^] related to the size of the particles. When *F*_*n*_ ≥ *F*_*n*,*max*_, the bond breaks and a tensile crack (mode I) occurs at the bond location.

In the shear direction, *F*_*s*_ is the driving force and is computed incrementally as:

$$F_{s}=F_{s,t-\Delta t}+k_{s}*\Delta u_{s}$$ (4)

with k_s_, the shear stiffness computed as *k*_*s*_ = *P* * *k*_*n*_, with *P* as a constant to define between the limits of 0 < *P* < 1. Δu_s_ is the relative incremental displacement and *F*_*s*,*t−Δt*_ is the shear force at the previous timestep. The maximum acceptable shear force, *F*_*s*,*max*_ is defined using a generalized Mohr-Coulomb criterion with the cohesion *c* (in Pa) and the friction angle *φ* (in °) as:

$$F_{s,max}=c*A_{int}+F_{n}*tan(\varphi )$$ (5)

When *F*_*s*_ *≥ F*_*s*,*max*_, shear failure develops, and a shear crack (mode II) occurs at the bond location. Due to the dynamic formulation of the method, global non-viscous damping is used to dissipate kinetic energy and facilitate the simulation under quasi-static conditions. All simulations in this study were performed with a damping coefficient equal to 0.4. Furthermore, the details of formulations and their more extensive derivations given above can be followed in Scholtès and Donzé [54] as well as in Dinc and Scholtès [44].

### Model parameter calibration

In order to represent the macro mechanical properties such as *UCS*, *UTS*, *E*, and *v* and the failure envelope of andesite, six microparameters (*Y*, *P*, *t*, *c*, *φ*, and *N*) defined in the previous section have to be calibrated to generate a numerical model sample (assuming that the rock is homogeneous and isotropic). Each parameter depicts a specific macro property of rock. For instance, *Y* controls Young’s (tangential) modulus (*E*) of the rock material and *P* as the ratio of *k*_*n*_*/k*_*s*_ affects the Poisson’s ratio (*ν*) that are obtained from the uniaxial and triaxial compressive and direct tensile test simulations. t is the tensile strength of the particles controlling the *UTS* of the model sample. c is the cohesion influencing the *UCS*. *φ* controls the slope of the failure envelope and is directly obtained from triaxial compressive test simulations. *N* is defined based on the ratio of *UCS*/*UTS* before the simulation starts. The *γ*_*int*_ provides to link particles with the contact ones as well as another particle in its neighborhood. When the interaction range increases, the grain interlocking ascends and the coordination number (*N*) increases (see [54]) for details).

The calibration process was driven by the simulation of laboratory tests such as the uniaxial compressive, triaxial compressive, and direct tensile strength tests. The three-dimensional numerical samples with dimensions of 1 x 2 x 1 model units were subjected to these test simulations, running under the same stress conditions in the laboratory. Each sample consists of 10,000 particles. For all simulations, the loading rate (velocity) was selected as 0.025 m/s based on the results of the preliminary sensitivity analysis. In the uniaxial compressive test simulations, the loading is applied to the model sample through two rigid and frictionless walls, taken placed at the top and bottom surfaces. (Fig 2a). In the triaxial compressive test simulations, there are six rigid frictionless walls (Fig 2b). When the relevant confining stress is reached, the top and bottom walls are moved vertically at a constant strain rate and the confining stress is controlled by adjusting the lateral wall positions. In tensile test simulations, the particles placed at the top and bottom boundaries of the sample move in opposite directions along the vertical axis. The velocity is applied through these boundary particles (Fig 2c). All models were assumed to have no pre-existing discontinuities and the microcracks were induced in the model samples during the loading. The calibration process was repeated until the macro mechanical properties of the models match those of the andesite. The microparameters adjusted during this process are given in Table 2.

**Table 2:** Microparameters of the calibrated DEM model.

Parameter	
**Elastic Modulus *Y* (GPa)**	16
**Stiffness Ratio *P* (-)**	0.5
**Interparticle Tensile Strength *t* (MPa)**	10
**Cohesion *c* (MPa)**	100
**Friction Angle *φ* (º)**	1
**Coordination Number *N* (-)**	10

## Results

### Model validation

In order to obtain the macro mechanical properties of andesite, first of all, laboratory tests were performed on the samples whose details are given in the section of Laboratory Experiments. Following this work, a series of uniaxial—triaxial compressive and uniaxial tensile test simulations were performed on 3D model samples until the mechanical behaviors of the numerical model represent the real ones.

**Fig 3:**
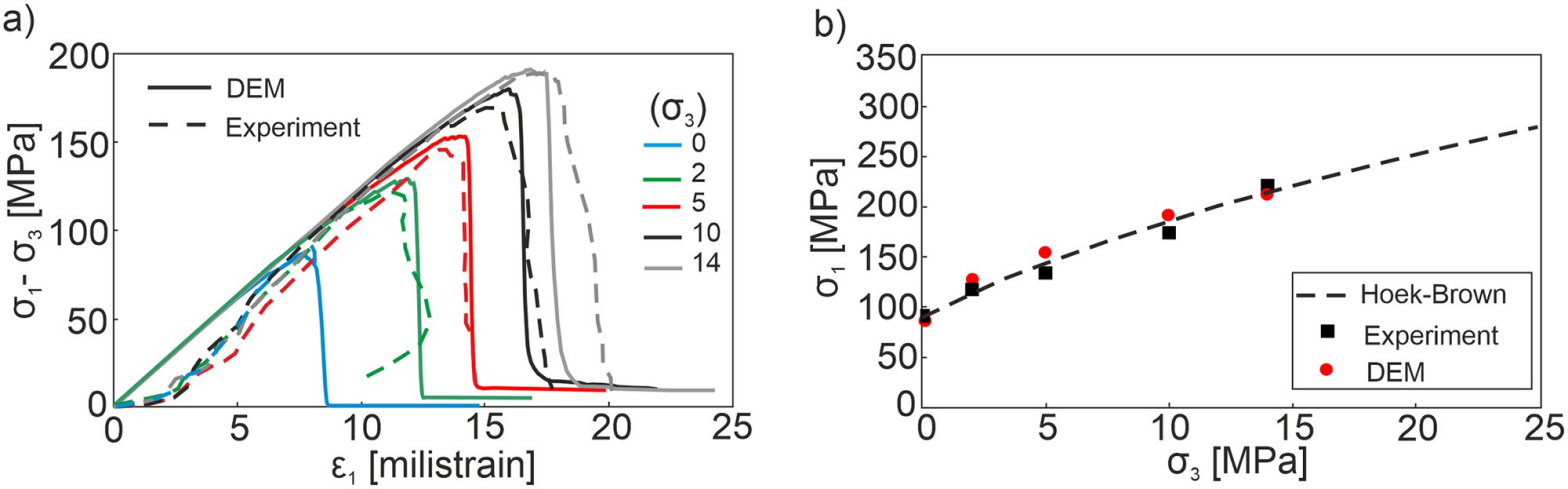
Triaxial compression test results under different confining stresses *σ*_*3*_ (0, 2, 5, 10, 14 MPa) derived from the 3D DEM model simulations and laboratory experiments. (a) Stress-strain curves (b) Hoek-Brown failure envelope.

The macro mechanical parameters predicted through the DEM simulations are listed in Table 1. One can see that good agreement has been achieved when comparing the predicted parameters from DEM simulations with those obtained from laboratory tests in terms of the stress-strain responses and failure envelopes (see Table 1 and Fig 3).

**Fig 4:**
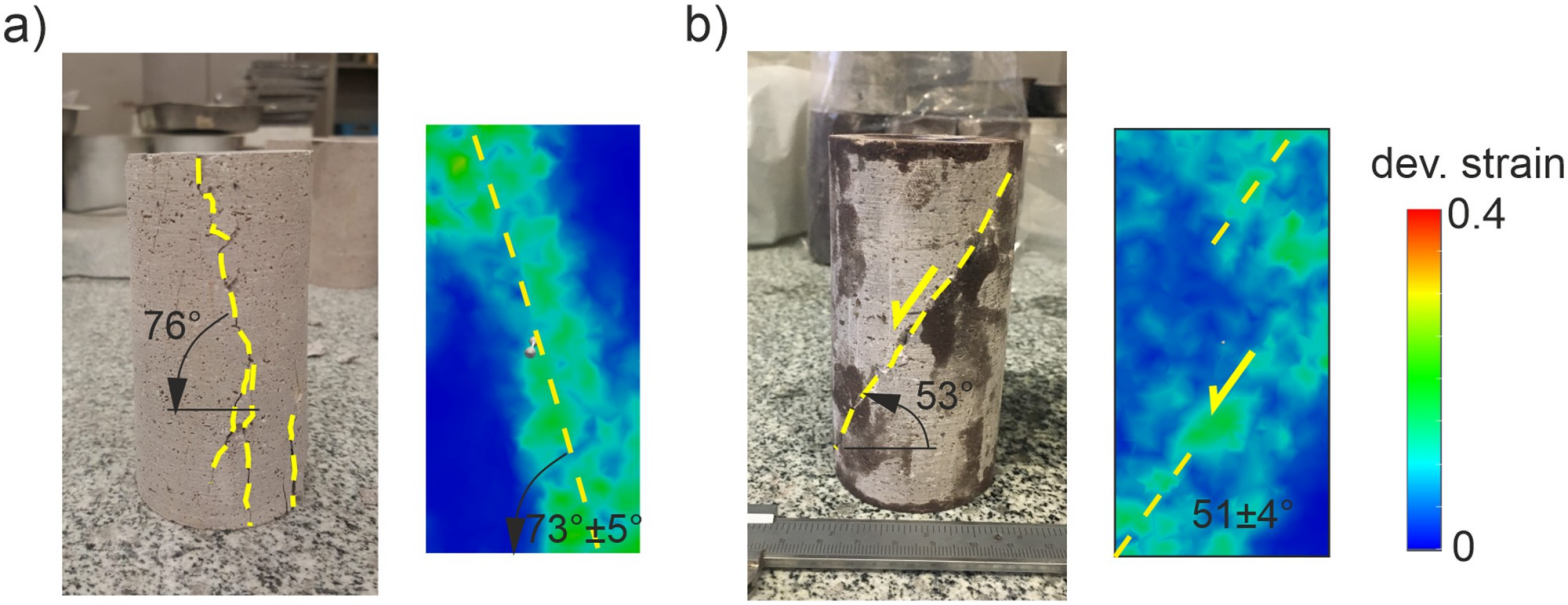
Macro-cracks in the laboratory samples (left) and strain field patterns in DEM model samples (right) after failure (a) Under uniaxial compressive loading condition (b) Under triaxial compressive loading (*σ*_*3*_ = 2 MPa) condition.

Moreover, the strain localization patterns were compared to the experimental crack patterns developed in the core samples for uniaxial and triaxial compressive stress conditions at the failure phase (Fig 4). As it can be clearly seen in Fig 4, the experimental macro-cracks and numerical strain field patterns have similar orientations that develop sub-parallel to the loading direction (≈ 73° dip angle from the horizontal axis) under uniaxial compressive loading. On the other hand, they are both oriented at approximately 51° dip angle from the horizontal axis under triaxial loading (*σ*_*3*_ = 2 MPa). Consequently, our preliminary results verify that the stress-strain response and cracking phenomena observed in the laboratory can be accurately captured by using the calibrated 3D DEM model sample. The numerical model has the ability to reproduce cracking and deformation processes whose details are provided in the following sections.

### Cracking analyses and calculation of strain fields

Since Bieniawski [6], researchers have focused on the macro mechanical behaviors of rocks under compressive stresses to reveal their relation to the induced micromechanical process. Herein we investigated the brittle rock responses in terms of progressive failure and examined the cracking process comprehensively in the model samples for two different confining stresses of *σ*_*3*_ = 0.1 MPa and *σ*_*3*_ = 2 MPa. It is worth noting that relatively low confining stresses (*σ*_*3*_ = 0.1 and 2 MPa) were selected for the discrete analysis even though the various confining stresses were tested in the laboratory experiments (see Fig 3). By doing so, the heavy catastrophic failure of the samples and so the complexity of the models are avoided.

**Fig 5:**
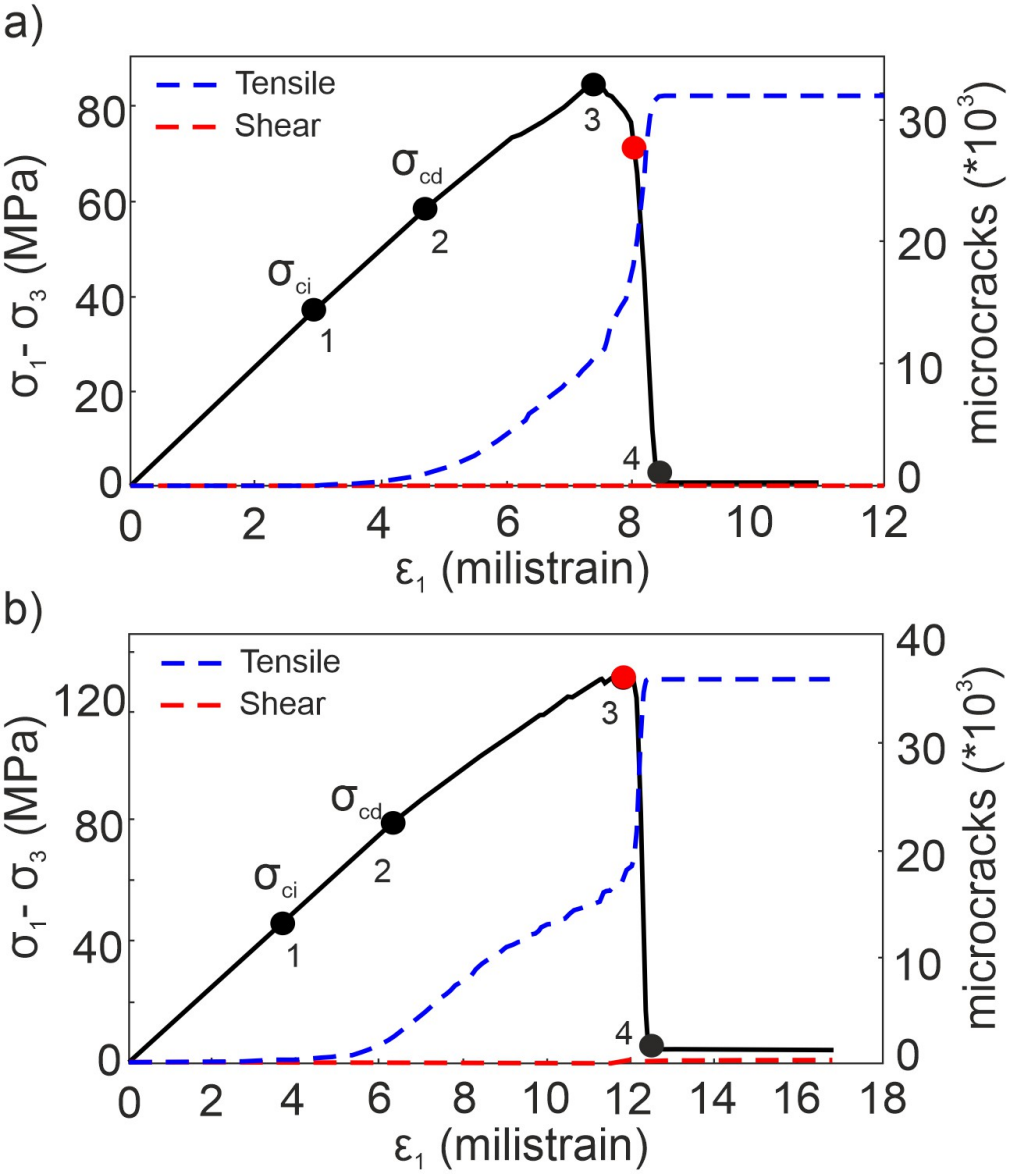
Stress-strain responses of andesite under triaxial loading conditions. (a) *σ*_*3*_ = 0.1 MPa (b) *σ*_*3*_ = 2 MPa (Black spots: (1) crack initiation stress *σ*_*ci*_, (2) crack damage stress *σ*_*cd*_, (3) peak stress, and (4) residual stress levels; Red spots: the stress levels of shear microcracks’ onset).

In both cases, the linear-elastic responses at the early stages of the loading transferred to the nonlinear trend with increasing axial stress (Fig 5). After the peak, a dramatic drop was observed in stress levels as a result of strain-softening into the numerical domain. During the simulations, the microcracks that emerged in the material pointed out four pronounced stress levels such as (1) crack initiation, *σ*_*ci*_; (2) crack damage, *σ*_*cd*_; (3) peak, and (4) residual [9, 12, 55–57]. Looking at the *σ*_*ci*_ and *σ*_*cd*_ stress thresholds, they are proportionally closer to the peak strength under the confining stress equal to 0.1 MPa (respectively 44% and 79% of the peak stress) than their positions on the stress-strain curve when *σ*_*3*_ = 2 MPa (respectively 34.6% and 61.5% of the peak stress). This consequence is correlated with an intense crack diffusion with increasing confining stress that induces the cracking process at the early phases of the loading. For both conditions, tensile microcracks (mode I) causing dilation in the perpendicular direction to the loading (called the Poisson effect) are the dominant driving mechanism of failure in model samples. It is because the numerical model represents brittle rock failure behaviors, interparticle (DEs) bonds preferentially break by tensile rupture rather than shear microcracking [54]. Even though the number of shear microcracks (mode II) developed during the simulations under confining stress of 2 MPa is more than the one that emerged under *σ*_*3*_ = 0.1 MPa, they are only 0.5% of the total crack population as a very limited number. Shear microcracks appear after the peak of the stress-strain curve under 0.1 MPa confining stress while they emerge once the peak is reached when applying *σ*_*3*_ = 2 MPa. That result obeys the principles of fracture mechanics such that the cracks nucleate at relatively earlier stages of the loading with the increase of confinement. Red spots in Fig 5 point out the shear microcrack nucleation stress levels.

**Fig 6:**
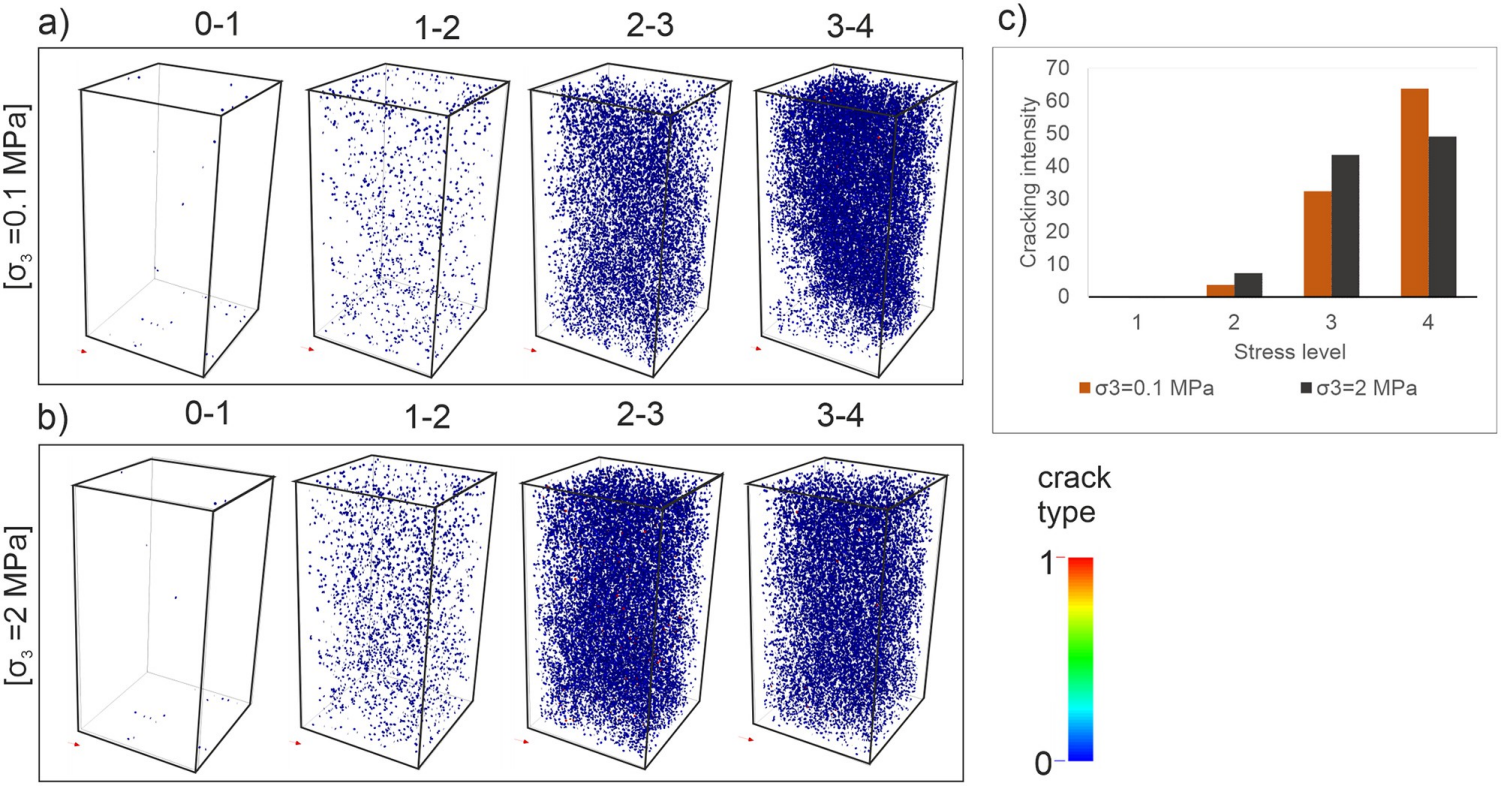
Spatial distribution of microcracks during the compressive test simulations. (a) *σ*_*3*_ = 0.1 MPa (b) *σ*_*3*_ = 2MPa (c) Cracking intensity for four stress levels (plotted in Fig 5).

Since the precursory signals of the final macro-crack pattern emerge before the ultimate rock strength is reached, it is crucial to predict this critical preceding part of the rock stress-strain response [15–17, 19, 37, 58, 59]. In this study, the spatial distribution of microcracks was grouped for four stress phases previously presented in Fig 5 (0–1; 1–2; 2–3, and 3–4) to analyze how the microcracks accumulate and indicate oncoming damage in the model samples. Correlating the elastic-linear material responses for the early stages of the loading presented above, the nucleation of the microcracks is not noticeable enough before the stage 1–2 (Fig 6). At the stage 1–2, the amounts of cracks are still very less, only 3.66% and 7.23% of the total number under the confining loadings of 0.1 and 2 MPa respectively (Fig 6a and 6b). As yet, it is difficult to estimate the final form of rock failure. Once the stress-strain curve deviates from its linearity (see Fig 5), the number of microcracks increases significantly and they diffuse intensely into the model sample (stage 2–3). That causes a decrease in material stiffness, and hence a significant decrease in inherent cohesion as well. The propagation and subsequently localization of the microcracks predominantly start in this phase and so the precursory signals of a macro-crack are manifested herein. Following the peak (stage 3–4), the stress drops dramatically, and the nucleation, propagation, localization, and coalescence of microcracks synchronously accompany each other. The friction takes the control of the damage in rocks. The microcrack population in the case of σ_3_ = 0.1 MPa (63.80%) is more than the accumulation in the other case (49.13%) at the stage 3–4. The majority of the cracking process is observed at this stage for both loading conditions (Fig 6c). Furthermore, one can observe in Fig 6 that confining stress has a significant effect on the rock deformation process [9, 10, 12, 14]. An increase in its value leads to greater strain and microcracks at the pre-failure stage (see Fig 5). From this result, it appears that the intensity of the damage regarding strain localization depends on the confining stress, hence the depth of the relevant rock.

## Discussion

**Fig 7:**
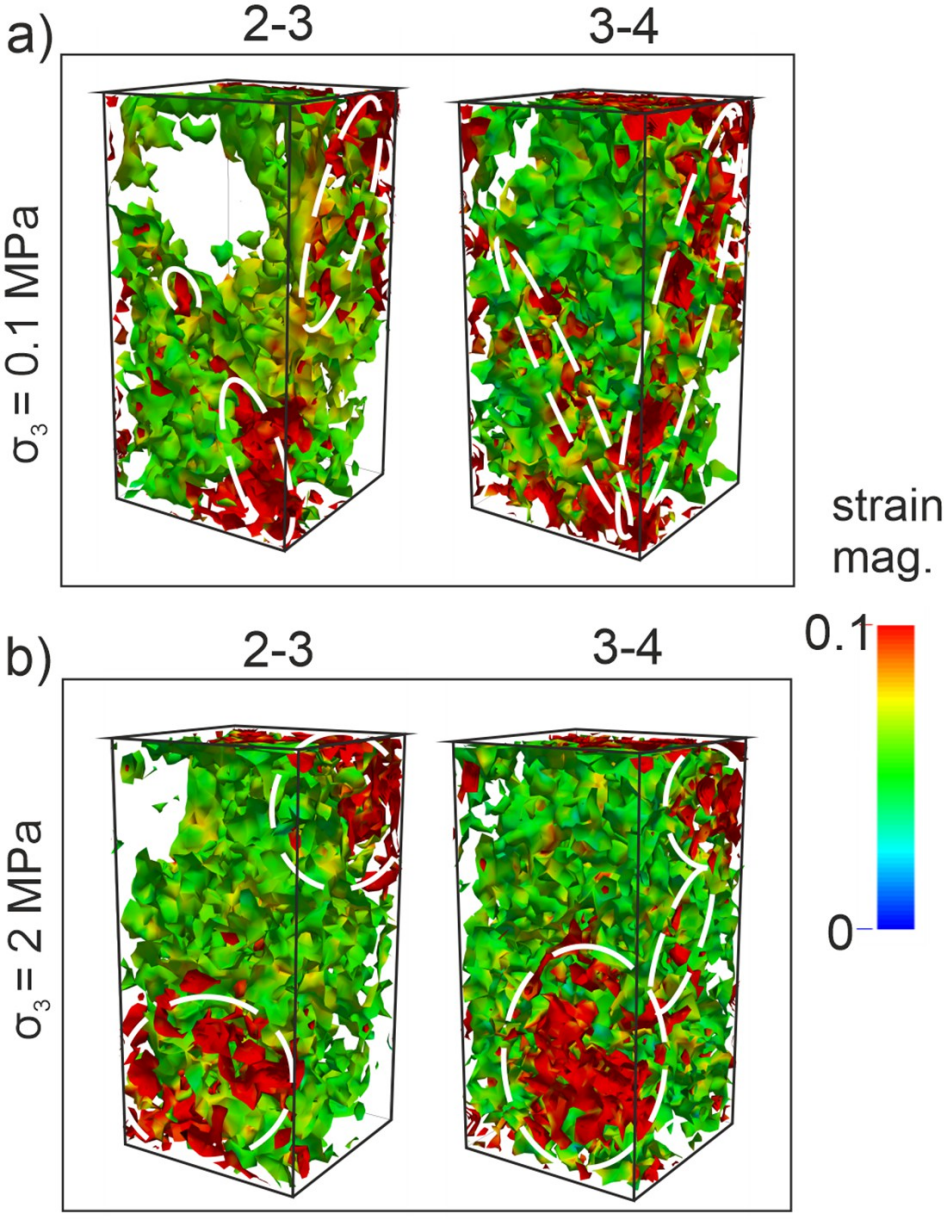
Strain fields computed between (2–3 and 3–4) in the 3D DEM simulations. (a) *σ*_*3*_ = 0.1 MPa (b) *σ*_*3*_ = 2 MPa.

**Fig 8:**
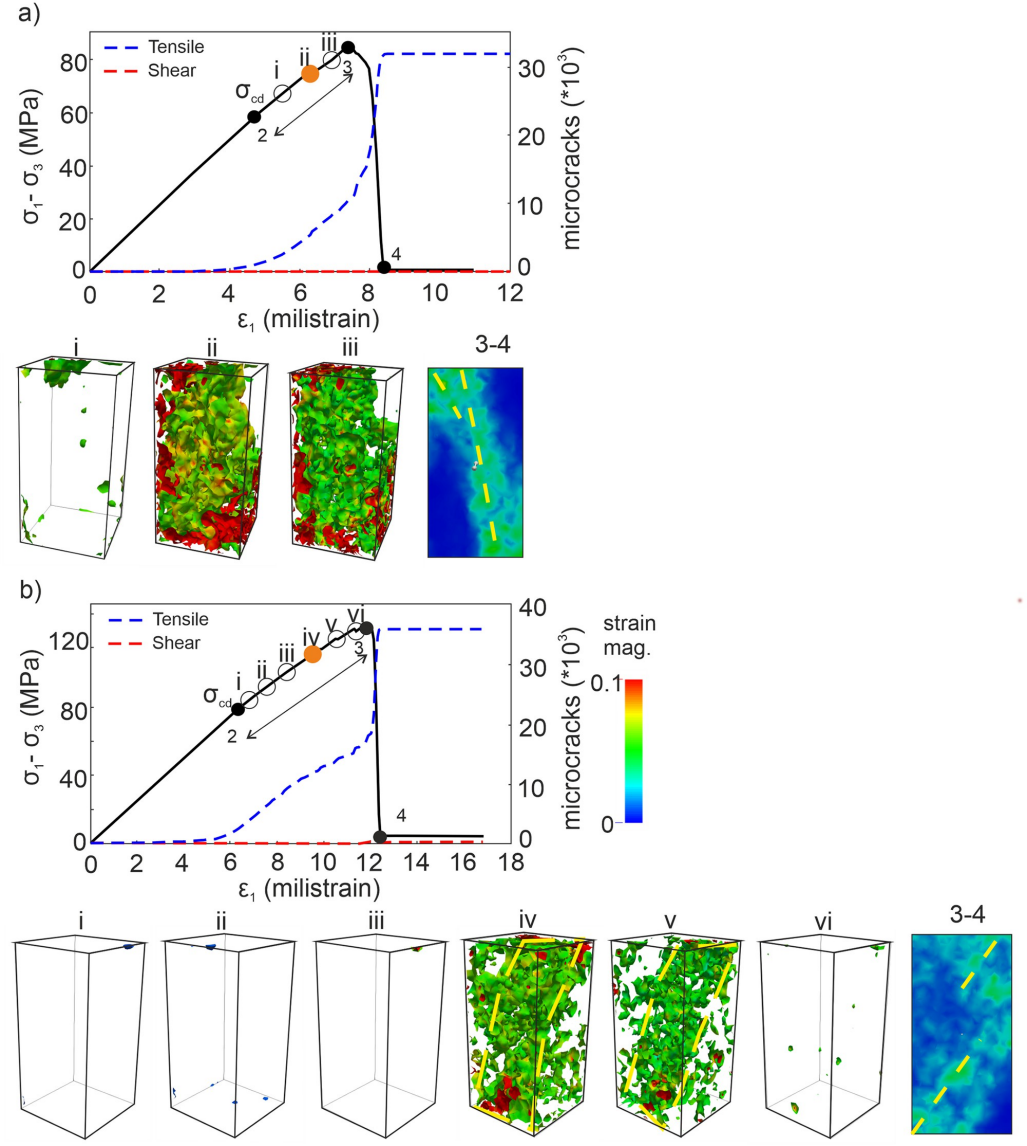
Stress-strain responses and incremental strain fields computed for different substages that divided along the stage 2–3 in the DEM analyses. (a) *σ*_*3*_ = 0.1 MPa (b) *σ*_*3*_ = 2 MPa.

The results of the analyses presented above show that the microcracking process can be taken into consideration in detail for the stage 2–3 to detect the origin of the rock damage observed at the stage 3–4. Therefore, the deviatoric strain fields were calculated for these phases in the DEM analyses (Fig 7). The directions and locations of strain fields might highlight the type or form of damage mechanisms regarding microcracking [39, 40, 41, 44, 60]. Looking at Fig 7a, the strain fields at the stage 2–3 present relatively sub-parallel oriented localization zones with respect to the σ_1_ as being well correlated with the cracking orientation concept proposed by Peng and Johnson [7]. Therefore, these zones could be considered as the signals of a splitting failure driving under extensional mechanisms [61] (Fig 7a, stage 3–4). When *σ*_*3*_ = 2 MPa, in Fig 7b, the strain fields at the stage 2–3 align predominantly with an inclination angle according to the loading direction which indicates most likely a slippage failure trend [37]. The linkage of the localization zones that appeared at the bottom and top of the model sample also forms the subsequent size of a macro-crack at stage 3–4 as well as stabilizes the final damage orientation as a characteristic property (Fig 7b). That finding also obeys the size effect law proposed by Bažant and Chen [59]. In addition, all computations in these analyses are consistent with the strain localization measurements under the compressive loading conditions studied by Baud et al. [18]; Louis et al. [33]; Zhang et al. [34]; Desrues and Ando [35] thanks to their descriptions of precursory strain fields. Nevertheless, the accurate stress level for the initiation of strain localization in these studies has not been addressed clearly. Therefore, the strain fields developed during the stage 2–3 were computed and recorded for every 10,000 iterations, to reveal directly when the damage zones initiate in the model samples (Fig 8). It is worth noting that when the iteration number is less than 10,000 there is not a remarkable crack accumulation in the model sample and the strain does not localize significantly. Therefore, one substage interval was chosen to correspond to 10,000 iteration numbers in the computations.

For the case of *σ*_*3*_ = 0.1 MPa, the interval belonging to the stage 2–3 on the stress-strain curve was divided into three substages shown as (i), (ii), (iii) in Fig 8a. The length of the interval of this stage is longer in the case of *σ*_*3*_ = 2 MPa due to the relatively higher confining stress [22, 24, 62, 63]. Thereby, the stage 2–3 of the stress-strain curve was now divided into six substages presented as (i), (ii), (iii), (iv), (v), and (vi) in Fig 8b.

The σ_cd_ values were detected as 61.50% and 67.44% of the relevant peak stress for the cases of *σ*_*3*_ = 0.1 MPa and *σ*_*3*_ = 2 MPa, respectively. The percentages obtained in this study are within the range of the σ_cd_ /σ_peak_ ratios for the low-porosity rocks suggested by Xue et al. [64]. Once the *σ*_*cd*_ values are reached, the microcracking intensity dramatically increases [44, 48, 65] but the localization zones of microcracks that represent the final macro-crack pattern have not developed within the model samples yet. In other words, this means that the indicators of the macro-crack can still not be observed whenever the *σ*_*cd*_ is reached [40]. When the dominant damage in a rock characterizes by tensile cracks, it is hard to observe the crack localization in specific regions of the sample because of low energy consumption, and energy dispersion is not concentrated [14]. Thus, we detected the exact points on the stress-strain curve where the indicators precisely appear along the stage 2–3 as representative patterns by computing the strain fields through the DEM analyses.

As one can clearly see in Fig 8a, the strain fields at the substage (i) do not give enough information regarding accelerated damage in the model sample. Directly following, evident indicators of the strain localization zones form at the substage (ii), corresponding to 87.2% of the peak stress. In the other case, looking at Fig 8b, the precursory signals have not been configured during the first three substages (i, ii, iii). Precise development of the localized zones onsets at the substage (iv), corresponding to 88.46% of the peak stress. These results show that the descriptive and representative precursory signals of a macro damage zone (macro-crack) announce approximately 88% of the relevant peak stresses under low confining stress conditions, similarly to the experimental observations of Cheng et al. [21] They determined that the precursory stress levels of microcracking are very close to the peak stress (at least ≈ %80 of ultimate strength) during the AE measurements on sandstone samples. In addition, McBeck et al. [40] also detected that the maximum strain localization in various rock types as being representative of the final system-size failure occurs near 90% of the failure stress in the X-Ray tomograpgy images.

Orange spots in Fig 8 point out the exact stress levels of these precursory strain fields of the macro rock damage detected in this study.

## Conclusions

The target of this study is a comprehensive assessment of the precursory damage mechanisms regarding microcracking in brittle rocks. For this purpose, we performed a series of laboratory tests on the andesite rock samples to obtain their macro mechanical parameters. These parameters were then used in the calibration process of the three-dimensional numerical model samples generated based on the DEM. The damage localization zones were examined through discrete analyses by calculations of the deviatoric strain fields in the 3D model samples. The main conclusions can be summarized as follows:

1. Ankara andesite as characterizing brittle rock responses was chosen as the material of this study and its macro mechanical parameters (*UCS*, *UTS*, *E*, and, *v*) were determined in the laboratory. The orientations of the macro-cracks developed under uniaxial–triaxial compressive loading conditions were measured for a comparison with the numerical model predictions.
2. The stress-strain responses, failure envelope, and failure patterns derived from the DEM analyses were in good agreement with the experimental measurements that verify an accurate calibration process of 3D numerical models. The numerical model can reproduce detailed cracking and deformation processes in rocks.
3. The spatial distribution of microcracks was classified for four stress levels such as the *σ*_*ci*_, *σ*_*cd*_, and peak to detect the intensity of the damage during different stages of the triaxial compressive loading. The result reveals that the increasing confining stress accelerates the damage’s intensity regarding microcracking at the pre-failure stage. 36.2% of the total number of microcracks are observed herein for *σ*_*3*_ = 0.1 MPa, whereas it is 50.87% when *σ*_*3*_ = 2 MPa.
4. The DEM analyses suggest that prior to failure, the sub-parallel oriented (≈15°) strain fields with respect to the σ_1_ indicate a splitting failure for relatively low confining stress (*σ*_*3*_ = 0.1 MPa). On the other hand, the strain localization zones develop with an inclination angle (≈39°) to the σ_1_ that displays a subsequent slippage failure for the confining stress of 2 MPa.
5. The descriptive and representative precursory signals of a macro damage zone (macro-crack) in brittle rocks emerge at approximately 88% of the peak stress under low confining stress conditions. This insight showed that the representative strain field zones of failure are not observed as soon as the *σ*_*cd*_ is reached in brittle rock.

Overall, the 3D DEM analyses provide a comprehensive framework for further studies aiming at linking microscale interactions to the complex macroscopic failure phenomena observed in nature. The perspectives of this work would be to study the cracking mechanisms under relatively higher confining stresses as well as consider the pre-existing microcracks in the discrete analyses. In addition, the effects of model size on damage localization need to be investigated in the future since the thickness of the strain localization zone is controlled by the dimensions of the sample that make them scale-dependent structures. These would be of great interest for a better description of precursory strain fields in brittle rocks.
